# Improving health promotion through central rating of interventions: the need for Responsive Guidance

**DOI:** 10.1186/s12961-017-0258-9

**Published:** 2017-11-23

**Authors:** Maarten Olivier Kok, Roland Bal, Caspar David Roelefs, Albertine Jantine Schuit

**Affiliations:** 1 Erasmus School for Health Policy and Management, Erasmus University Rotterdam, Burgemeester Oudlaan 50, 3062 PA Rotterdam, The Netherlands; 2 Amsterdam Public Health Institute, VU University, Amsterdam, The Netherlands; 3 Science and Society, Faculty of Mathematics and Natural Sciences, University of Groningen, Groningen, The Netherlands; 4 National Institute for Public Health and the Environment, Bilthoven, The Netherlands

## Abstract

**Background:**

In several countries, attempts are made to improve health promotion by centrally rating the effectiveness of health promotion interventions. The Dutch Effectiveness Rating System (ERS) for health promotion interventions is an improvement-oriented approach in which multi-disciplinary expert committees rate available health promotion interventions as ‘theoretically sound’, ‘probably effective’ or ‘proven effective’. The aim of this study is to explore the functioning of the ERS and the perspective of researchers, policy-makers and practitioners regarding its contribution to improvement.

**Methods:**

We interviewed 53 selected key informants from research, policy and practice in the Netherlands and observed the assessment of 12 interventions.

**Results:**

Between 2008 and 2012, a total of 94 interventions were submitted to the ERS, of which 23 were rejected, 58 were rated as ‘theoretically sound’, 10 were rated as ‘probably effective’ and 3 were rated as ‘proven effective’. According to participants, the ERS was intended to facilitate both the improvement of available interventions and the improvement of health promotion in practice. While participants expected that describing and rating interventions promoted learning and enhanced the transferability of interventions, they were concerned that the ERS approach was not suitable for guiding intervention development and improving health promotion in practice. The expert committees that assessed the interventions struggled with a lack of norms for the relevance of effects and questions about how effects should be studied and rated. Health promotion practitioners were concerned that the ERS neglected the local adaptation of interventions and did not encourage the improvement of aspects like applicability and costs. Policy-makers and practitioners were worried that the lack of proven effectiveness legitimised cutbacks rather than learning and advancing health promotion.

**Conclusion:**

While measuring and centrally rating the effectiveness of interventions can be beneficial, the evidence based-inspired ERS approach is too limited to guide both intervention development and the improvement of health promotion in practice. To better contribute to improving health promotion, a more reflexive and responsive guidance approach is required, namely one which stimulates the improvement of different intervention aspects, provides targeted recommendations to practitioners and provides feedback to those who develop and rate interventions.

## Background

Increasingly more countries are putting systems in place to assess the quality of health promotion interventions, with the ultimate aim of contributing to better health promotion [1–5]. The idea of encouraging and steering improvement through central quality rating is inspired by the success stories of the evidenced-based movement [6, 7]. In a traditional evidence-based approach, the available evidence for the effectiveness of interventions is centrally rated, after which local users are encouraged to implement the most effective interventions [5]. In such an approach, interventions are viewed as transferable packages and quality is operationalised as the proven effectiveness of an intervention.

An evidence-based approach can provide important benefits [8]. Central rating of the effectiveness of interventions by experts can help health workers, policy-makers and others benefit from intervention development, research and appraisal work that has been carried out elsewhere and enhance the efficiency, effectiveness and legitimacy of health promotion [5, 6]. It can create an arena for the articulation of standards and the sharing and integration of knowledge and may thereby facilitate learning in the health promotion system [9].

While an evidence-based approach to health promotion seems promising and may yield substantial benefits, attempts to apply an evidence-based approach to lifestyle-related health promotion have faced many challenges [5]. Healthy behaviour depends on many factors that are deeply intertwined in a complex social context, which makes it difficult to assess and attribute effects to interventions, decontextualise them and reproduce similar ‘effective’ interventions elsewhere [10–12]. In addition to this intertwinement and multi-causality, it may take a long time before related changes in health are achieved, which further complicates the challenge of assessing effects and determining their relevance [1, 13, 14]. The promise of an evidence-based approach and the encountered challenges have fuelled a search for better ways of employing the central rating of effectiveness in health promotion [2, 4, 15]. An interesting attempt to apply the ideas of the evidence-based movement to health promotion is the Effectiveness Rating System (ERS) (also known as Recognition System). Since 2008, the ERS has been applied to lifestyle-related health promotion interventions in the Netherlands by the Centre for Healthy Living (in Dutch Centrum Gezond Leven) and partners [3]. One of the core tasks of the Centre for Healthy Living is to assess the quality of available – previously developed – health promotion interventions. In the ERS approach, intervention developers are requested to describe an available intervention according to a standardised format and submit it for rating. These interventions are then assessed by an ERS committee, consisting of health promotion research and practice experts. Local health promoting professionals are subsequently encouraged to use the interventions that have received the highest rating level. The potential benefits and reported challenges of applying an evidence-based approach to health promotion call for an analysis of how the ERS works in practice and the extent to which it actually contributes to improvement of health promotion.

In order to gain a better understanding of how the ERS works in practice, and to contribute to its optimisation, it is essential to take into account the ways in which existing structures, the parties involved and the procedures are embedded in larger regimes and systems. The central rating of the quality of interventions is not a goal in itself, but part of a strategy for improving health promotion in practice. In the ERS and this broader improvement strategy, a variety of actors (e.g. researchers, policy-makers and health promotion practitioners) play a role [3]. These diverse professionals function within their own regimes, such as science, politics and local health promotion, with their own incentives and accountability criteria [16]. Policy-makers must take into account legitimacy and social acceptability. Researchers are held accountable for their publications and health promotion practitioners are concerned with the feasibility and effects of health promotional activities in their specific local context [12]. The involvement of and reliance on actors from differing regimes has consequences for the ERS. The diverse professionals that are involved must be able to fulfil their function in the ERS, while simultaneously functioning in their own regime where the ERS may also fulfil a function for them. A good understanding of this interdependency is essential for those responsible for managing and optimising the ERS. Although a lot has been written about evidence-based health promotion, much less is known about this interdependency and the eventual contribution of central quality rating to the improvement of health promotion.

The aim of this study is to explore the actual functioning of the ERS and the perspectives of researchers, policy-makers and practitioners regarding its contribution to improvement in practice. We explored these questions from a pragmatist perspective and followed a case study design.

### The ERS

After several years of preparation, the Centre for Healthy Living was established in 2008 at the request of the Ministry of Health at the National Institute for Public Health and the Environment (Dutch acronym: RIVM). One of the core tasks of the Centre for Healthy Living was to develop and manage the ERS (which is also known as the Recognition system and described in detail elsewhere) [3]. In the ERS, developers of lifestyle oriented health promotion interventions are invited to describe their interventions according to a standard format and submit these for rating. Two committees (one for youth and one for adults) consisting of experts from science and local practice are charged with assessing the submitted interventions. Interventions can be recognised on three incremental levels of the so-called effectiveness ladder, namely (1) theoretically sound, (2) probably effective and (3) proven effective [3, 8].

Rating of ‘theoretically sound’ requires that the targets, strategy, preconditions and the process through which the intervention is supposed to impact health are described and reference is made to an established health behaviour change theory. The second level of ‘probably effective’ involves the additional requirement that the effectiveness be demonstrated in at least one methodologically strong study in the Netherlands, or three studies with lower validity. The highest level, ‘proven effective’, requires two methodologically strong Dutch studies, or one strong Dutch study combined with two strong foreign studies.

## Methods

### Study design and study population

This in-depth case study was part of a larger evaluation of the functioning of the ERS and its contribution to improving health promotion in practice, which was jointly developed by university-based researchers and the RIVM in the Netherlands. Data for this case study were collected by means of conducting semi-structured interviews and observing the assessment of 12 interventions in two different ERS committee meetings.

For a first series of interviews, we purposively sampled 15 of the 30 Municipality Health Services in the Netherlands based on size and geographical representation. Professionals involved in health promotion at these Municipality Health Services were approached for voluntary interviews. A second series of interviews was held with 17 purposively selected key-informants who were professionally related to, or involved in, the ERS. The aim was to sample influential stakeholders with a broad range of roles related to the ERS (e.g. committee members, members of advisory councils) in research (e.g. leading scientists in health promotion, research funding organisations), policy (e.g. in municipalities) and practice (e.g. health promotion practitioners). Participants were approached by telephone and by email. All people who were approached for this study agreed to participate.

### Interview guide development and the interviewing process

The topic list for the interviews was developed in two steps. A first topic list was developed in close consultation with Centre of Healthy Living staff and piloted in two interviews. After piloting, the order of the topics was changed and a final topic list was established. The topic list was flexible enough to be adapted to each type of interviewee (e.g. policy-maker, researcher, health promotion practitioner) depending on their specific role and expertise.

The interviews commenced with basic questions about the participants employment background and an exploration of the participants relation to the ERS (e.g. involvement in submitting interventions, the committees, indirect involvement as research funder, policy-maker). The interviews continued with open questions about the precise role of the interviewee in relation to the ERS and the role that the ERS potentially fulfils for the interviewee in their own regime. This was followed by exploring how participants expected the ERS to contribute to improvement in health promotion and their perspective on the functioning and specific problems of the ERS. The interviews concluded with cross-cutting questions that addressed particular themes or hypotheses that emerged from the earlier interviews. Interviews were held at the location where participants worked and lasted about an hour. The process of discussing and rating 12 interventions was observed and audio-recorded during two committee sessions.

An experienced university-based interviewer (MK) was involved in all interviews and the observation at both rating committee meetings. A second experienced interviewer was involved in about half the interviews in order to prevent interviewer bias. Permission was asked for recording the interviews and the recordings were transcribed verbatim. One interviewee did not want to be recorded and in one interview the equipment failed. Notes were taken during these interviews and they were typed up in more detail immediately afterwards.

### Data analysis

The observed committee meetings and all but two interviews were audio recorded and transcribed verbatim. After each interview and observation, a detailed summary was prepared by one of the interviewers, using the audio-tapes and notes taken during the interviews and observations. A data management assistant supported the researchers in transcribing the interviews and the recorded meetings and helped coding the data. MAXQDA was used to code the transcripts and notes. Some themes were identified in advance and others were derived from the data. After a first round of open coding, codes were checked independently by a second researcher, after which the coding and emerging themes were discussed. After a second round of coding, summaries were made for each topic by going through the detailed summaries of the interviews and observation notes and coded transcripts identifying themes using a constant comparative method of analysis [17]. Theme-specific summaries were then developed [18].

This study did not require ethics approval according to current Dutch law. Verbal consent to participate in the interviews, record the interviews and use the results for publication was obtained from all participants. Care has been taken to ensure that no comments can be traced back to an individual.

## Results

### Description of sample

In total, 53 participants in the Netherlands were interviewed for this study between August 2009 and August 2012. The participants had various primary professional positions, as members of government organisations (e.g. Ministry of Health, municipalities) (*n* = 7), researchers (*n* = 6), employees of the research funder ZonMw (*n* = 3) or health promotion practitioners in a Municipality Health Service (*n* = 37). Some participants had multiple secondary positions, such as members (*n* = 4) or chairs (*n* = 2) of the observed ERS committee, advisors to the Centre for Healthy Living (*n* = 8), the Ministry of Health (*n* = 9) or municipalities (*n* = 28), and contributing to research (*n* = 17) and intervention development (*n* = 26).

### Assessed interventions

**Fig. 1:**
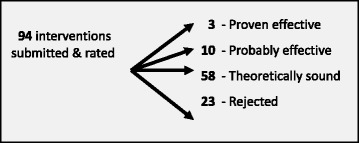
Interventions submitted, assessed and rated

Between their inception in 2008 and the end of 2012, the two ERS committees assessed a total of 94 interventions. Of the assessed interventions, 23 were rejected, 58 were rated as ‘theoretically sound’, 10 were rated as ‘probably effective’ and 3 were recognised as ‘proven effective’ (Fig. 1).

### The roles of involved actors in the ERS

We asked participants which role the involved professionals were expected to play in the ERS committees and in the broader improvement strategy of which it is part. Several participants who worked as policy-makers and health promotion practitioners pointed out that researchers and the scientific regime played a prominent role in the ERS. They said that the improvement strategy positioned effectiveness research as a necessary requirement for achieving better health promotion. Several health promotion practitioners pointed out that researchers played a key role in designing the tools and procedures of the rating system (e.g. the format for describing interventions, the criteria and rating levels). Furthermore, researchers chaired the ERS committees and many of the committee members were also researchers. Participants said that policy-makers were expected to contribute to the ERS improvement strategy by encouraging professionals to submit interventions for rating and requesting health promotion practitioners to use rated interventions. The independent Healthcare Inspectorate was expected to play a role by assessing the use of rated interventions in future inspections. Participants also pointed to the role of the research funding organisation, ZonMw, which required that recipients of funding had to submit interventions for rating. The final group of professionals that was expected to play a role in the ERS strategy were health promotion practitioners, who emphasised that, while they had limited influence in the ERS, a lot was expected from them. They were expected to be involved in effectiveness research, to describe and submit interventions for rating, and to implement those interventions that receive the highest rating.

### The role of the ERS for the actors involved

We asked participants which role the ERS was expected to fulfil for the different professionals that were involved in it. Health promotion practitioners and policy-makers pointed out that the ERS approach seemed very useful for researchers, as the strategy required the investment of large amounts of effectiveness research. In addition, the ERS anchored the role of research in both determining the rating criteria and in applying these in assessing interventions. Participants explained that the ERS could be useful for policy-makers as a source of information and as a means of legitimising decisions about health promotion. They further said that the overview of rated interventions allowed the research funder ZonMw to more easily target its investments in intervention research and development. Staff of the Healthcare Inspectorate said that the rating of interventions provided them with an instrument for examining the quality of health promotion. Researchers, policy-makers and staff of the Healthcare Inspectorate expected that the ERS could help health promotion practitioners in selecting the most appropriate interventions. While most health promotion practitioners were sceptical towards the ERS ratings, they often said that it encouraged them to reflect on their work.

### The role of the ERS in two improvement dynamics

We explored the perspective of participants on the aim of the ERS and the way it was intended to contribute to improving health promotion. Participants had different ideas about the roles of the ERS and used different terms to describe these roles. Some described its role simply as determining the effectiveness of interventions. Others described the ERS as an attempt to control local health promotion. The most often described roles were ‘stimulating of intervention improvement’ and ‘advising on intervention quality’ by providing an overview of available interventions and insight into the effectiveness and other characteristics of interventions. We asked participants to describe how, according to them, the ERS was intended to contribute to improving health promotion. Their responses showed that the ERS was intended to contribute to two separate, but related, improvement dynamics, wherein the first dynamic led to improvement of available interventions and the second dynamic led to improvement of health promotion in local practice.

### First role of the ERS identified by participants: stimulating the improvement of interventions

Participants consistently said that the ERS was intended to stimulate the improvement of available health promotion interventions. While participants generally agreed that the ERS led to some positive developments, most of them said that it faced too many limitations to effectively encourage and steer improvement of the reservoir of available interventions.

In the ERS, the first rating level requires that an intervention is thoroughly described. Participants often emphasised how important it was that the ERS encouraged the accurate and thorough description of interventions and explained that it promoted reflection, learning and improvement with respect to available interventions.

“*When we wanted to submit the intervention, we were confronted with questions, things we hadn’t thought through well enough. What exactly was the theory that we applied? What evidence is available for that theory? You’re constructing and reconstructing that when you submit an intervention. You identify blind spots and have to fill those in. It is a way in which you certainly increase quality and improve substance. That’s the benefit. I can certainly see a learning effect.*” (Health promotion practitioner/policy advisor)

Some researchers were worried that rating would only result in better descriptions, rather than improving interventions.

“*One of the things I am concerned about is that only the descriptions of interventions are improved. That isn’t what this is all about. We want them to improve the intervention itself. They think that when they describe it a little nicer, we will all of a sudden like their intervention.*” (Researcher/committee member)

### Problems with effectiveness and the effectiveness ladder

The designers of the Effectiveness Ladder explained how the rating levels were meant to create a trajectory that stimulated improvement of available interventions. While participants supported the idea of using rating to stimulate intervention improvement, they questioned whether a hierarchy that focuses only on proven effectiveness was the most suitable approach. Participants argued that the stepwise rating on the effectiveness hierarchy was both too ambitious and too limited, and therefore unsuitable for guiding the process of intervention improvement. The most frequently expressed criticism was that the highest level in the ERS (‘proven effective’) was unattainable for most interventions in health promotion because the criteria were too demanding. Three specific issues related to effectiveness were raised, namely (1) shared norms for relevant effects were lacking, (2) there was uncertainty about how the effect of health promotion should be studied and (3) the ERS was not suitable for complex intervention processes.

#### The lack of shared norms for relevant effects

A first issue that was raised was that norms or standards for relevant effects were lacking (e.g. what percentage of a target population should lose how much weight at what point in time before an anti-obesity intervention should be rated as effective?). The interviews and observations revealed that the absence of these norms were a constant source of questions and discussion when rating interventions. While participants considered effectiveness important, they found it difficult to describe which effects were minimally required before an intervention was recognised as ‘effective’. For those not partaking in the ERS, it was unclear which standards or norms were used to determine which effects were relevant and how much ‘effect’ was required before an intervention was rated as ‘effective’. When we asked participants to describe such standards or norms, they either refused or found it very difficult and pointed to other stakeholders to do so. The Healthcare Inspectorate argued that it was up to the ‘field’, while health promotion practitioners mostly pointed to researchers and the rating committees, but also emphasised that citizen preferences should be taken into account. Committee members indicated that norms for the relevance of effects were seldom clear and were often constructed ad hoc while assessing an intervention.

“*I find it a difficult process that is increasingly getting more complicated. So many factors play a role and the weighting method is not unambiguous. I sometimes think, now we find this one theoretically well founded and then that one goes further and not that other one, and how they differ, I do not know. The criteria are abstract. In such a committee meeting a kind of process emerges whereby a decision is ultimately made. Sometimes the balance tilts one way or the other, while I think, if it had been in a different context or with slightly different committee members around the table, it just might have tilted the other way and would be proven effective.*” (Health promotion practitioner/committee member).

The difficulties resulting from a lack of norms for relevant effects became obvious during the observations of the rating committees. One intervention that did not have the expected effect size, but did show some slight effects, raised much discussion. In the approach of the ERS, intervention submitters had to set specific objectives, including relevant effects, before an intervention was studied. When research failed to demonstrate the anticipated effects, the rating committee decided it could not rate the intervention as ‘probably effective’. However, some committee members were reluctant to dismiss the intervention because, even though the effects were smaller than anticipated, they could still be relevant. Finally, it was decided that, in order to be eligible for rating, the intervention needed to be resubmitted with adjusted objectives, aiming for less ambitious effect sizes.

#### Uncertainty about how health promotion has effect

A second challenge was that there were continuous discussions and insufficient agreement about how health promotion interventions had effects and when and how such effects could be established. Several participants, especially researchers and health promotion practitioners, criticised the idea of, and focus on, separate interventions that should lead to linear, directly connected and easily demonstrable effects. The interviewed health promotion practitioners and researchers often emphasised that healthy behaviour is highly contextual and influenced by a large number of evolving factors. They argued that different health promotion activities together may affect health trends, but that such changes are not easily attributable to individual interventions. They suggested that more research is needed into how different components or mechanisms together lead to health and how research and monitoring can best be deployed to contribute to health promotion.

#### The ERS is not suitable for multi-component intervention processes

A third problem that was described was that the ERS was not suitable for multi-component intervention processes. Several participants argued that the ERS was only suitable for rather simple, well-defined interventions with a clear beginning and end point that were aiming at changing behaviour at the individual level. Health promotion practitioners and researchers argued that, in lifestyle-related health promotion, the most effects were expected from intervention programmes that contained different integrated components and which were preferably co-created in practice. The strength of such interventions seemed to result from the joint working of components, the engagement of local actors and the responsiveness to local circumstances. Participants argued that the quality of such intervention processes depended not on the protocol, but on the way the process was run, the competencies and motivation of those involved, and the learning throughout the intervention process. These intervention processes were not easily studied by simple before and after measurements and would be different when they were implemented at other times and places.

“*The rating system is aimed at structured, orderly, sequential interventions, rather similar to what is common in the medical field,* […] *while in health promotion we need to take into account changes in the environment that are less easily testable, so achieving ‘proven effectiveness’ for the interventions that are probably the most effective could be more difficult than it is for interventions that focus on one detail but will have less impact on population health.*” (Researcher/committee member)

In interviews with committee members and during the observed committee sessions, it was argued that the rating criteria had to be adapted according to the kind of intervention. Some suggested a more relative approach in which the most suitable study design was first determined and then the strength of evidence was discussed.

### Other limitations of the ERS rating strategy

Besides the specific problems and challenges with effectiveness, participants described five other problems that were limiting the contribution of the ERS to the improvement of available interventions, namely (1) necessary learning processes were ignored, (2) improvement of applicability aspects was not encouraged, (3) a mechanism for continuous feedback and improvement was lacking, (4) integration of knowledge was not encouraged, and (5) the necessary research funding was lacking.

#### Necessary learning processes are ignored

Participants from different constituencies argued that the Effectiveness Ladder was too limited because it only encourages research into effectiveness, while other learning processes necessary for improving interventions were ignored. Health promotion practitioners and researchers said that more had to be learned about how interventions were adapted and realised in local practice, how intervention mechanisms worked in context, the costs and competencies required for interventions, and the experiences of health promotion practitioners with interventions. Some researchers and health promotion practitioners argued for promoting a variety of organised learning processes (e.g. cost analysis, process monitoring) and the need to share these lessons through a central platform.

“*We have learned a lot from monitoring and reflection with stakeholders during the intervention process. Those lessons should be compiled somewhere and shared and added to the intervention.* […] *research should also say something about the elements of the intervention theory and about the working principles in it, and what is also needed is more emphasis on the contextual requirements and local applicability. So that means that you do need to set some additional requirements*.” (Researcher/committee member)

#### Improvement of applicability aspects is not encouraged

Health promotion practitioners argued that the ERS did not encourage the actual improvement of applicability aspects of interventions such as the costs, adaptability and context requirements. They pointed out that one of the three interventions that were recognised as ‘proven effective’ was never used because it seemed impossible to implement in practice. Some health promotion practitioners said that this illustrated that the Effectiveness Ladder was too limited to stimulate the required improvement of interventions. They argued for a system that should also stimulate making interventions cheaper, more flexible, less demanding and easier to implement.

“*They are only talking about effectiveness, and then provide you with an impractical intervention that demands a lot from intermediaries and from us and costs a lot of money. We need something that is easily applicable and efficient and attractive for participants. That must be improved as well; effectiveness alone makes no sense*.” (Health promotion practitioner)

#### A mechanism for continuous feedback and improvement is lacking

Participants who were closely involved with the ERS argued that the improvement of available interventions should be a continuous process. They described that, in the past, interventions were often developed by universities and disappeared after the project funding ran out. Participants were pleased that the ERS required that each intervention had an ‘owner’ who remained responsible for it. They argued that feedback from the rating process and from the implementation of interventions should be used to continue intervention improvement over time. They proposed an iterative approach to rating and financial incentives to stimulate continued improvement.

#### Integration of knowledge is not encouraged

Another concern that was raised by both researchers and health promotion practitioners was that the ERS did not encourage the integration of knowledge about health promotion. Moreover, some were worried that the focus on single interventions would actually lead to fragmentation of knowledge. Members of the rating committees said that they had the impression that they regularly assessed interventions that were substantively very similar. Some researchers argued that a classification system or taxonomy could be useful to prevent overlap, achieve efficiency and stimulate collective learning for health promotion. Such a classification or taxonomy of mechanisms would make it possible to compare interventions, integrate knowledge and prevent overlap and fragmentation. Participants pointed to taxonomies that were being developed and could possibly be used in the near future.

“*We should not strive for evidence-based, but theory-based health promotion, and with this I mean really that you shouldn’t be testing every small intervention in every singular context because then we will need ten times the budget and then we are spending it all on testing and not on implementing. You must identify overarching principles that appear to be effective in certain contexts*.” (Researcher/Committee member)

#### The required research funding is lacking

A final issue that participants raised was that only a fraction of the funding that was required for assessing the effectiveness of all submitted interventions was available. The ERS required two positive effect studies before an intervention could be rated as ‘proven effective’. Researchers pointed out that they were unlikely to receive funding to investigate the effectiveness of an intervention for a second time. The research funder ZonMw stated that it could fund only a few effectiveness studies each year, while hundreds of studies were required for the ERS strategy.

“*You already know from the beginning you are never ever going to achieve that level because you really don’t have the resources to finance such research, so in itself I think the criteria are very scientifically correct, but it is almost impossible to arrive at that highest level*” (Employee of research funder ZonMw)

Some participants who were involved in the ERS committees wondered how many new studies were required when one of the earlier studies showed no effect, and asked how many negative studies were required before an intervention was formally recognised as ‘proven not effective’. Participants generally agreed that the ERS strategy required a lot more research funding than what was available and worried that the number of interventions that could be rated as ‘proven effective’ would remain very limited in the years to come.

### The second role of the ERS identified by participants: contributing to improving health promotion in local practice

The second dynamic to which the ERS was intended to contribute was the improvement of health promotion in practice. Several participants said that, over time, the ERS could contribute to better health promotion by making it easier for practitioners to select the most appropriate interventions. Some health promotion practitioners said that they expected that the more thorough descriptions of interventions would facilitate their transfer and improve their use. Most participants considered it likely that, in the long term, the improvement of the available interventions would also contribute to better health promotion in practice. Besides these hopeful expectations, participants pointed to three limitations of the ERS that were hampering its contribution to better health promotion.

### The impression that health promotion does not work

Several participants, especially health promotion practitioners and policy-makers, were concerned that the ERS was leading to the perception that health promotion does not work and would thereby legitimise cutbacks. They pointed out that most interventions that were put into practice had not been thoroughly studied and only three of the hundreds of interventions currently used were formally recognised as ‘proven effective’. While the designers of the ERS stressed that the lack of ‘proven effectiveness’ should be used to lobby for more investments in research, several participants said that they were worried that the terminology used by the ERS was easily misunderstood and used by opponents of health promotion to legitimise reduced spending. Some health promotion practitioners and researchers pointed to a recent decision by the Minister of Health, who used the argument of ‘not proven effective’ to cut back on expenditure for national health promotion programmes.

“*The rating system can be used, or abused, to make prevention even less important, and that reduces the budget for research and prevention. You could also argue that when the lack of evidence for effectiveness is noted, but we decide that prevention is so important for public health, then it could also lead to more research budget to work towards more effective interventions.*” (Researcher/committee member)

### Other knowledge and descriptions required

Several participants argued that, to better contribute to the improvement of health promotion, more attention must be devoted to other kinds of knowledge and for further enhancing intervention descriptions. Health promotion practitioners were especially interested in the ‘story’ of how an intervention was realised and implemented, descriptions of how essential elements worked and the assumptions interventions made about local situations. To further improve their planning and decision-making, health promotion practitioners and policy-makers were also keen on better descriptions of the resources required for an intervention. Several participants pointed out that publications of effectiveness studies provided little insight into what was done to realise an intervention in a specific local context and eventually produce an effect. They emphasised that the format for intervention description used by the ERS should pay more attention to these aspects.

“*If I look purely from a local practice point of view at what is being submitted, it is not a practice story, it is not the story of how an intervention was carried out, what they encountered in practice, what the findings were of those who realized the intervention, what they themselves view as the strengths and weaknesses. What remains hidden is that grey area and all the knowledge that is not made explicit.*” (Health promotion practitioner)

In addition, health promotion practitioners explained that they regarded face-to-face meetings with intervention developers or experienced users more useful than reading intervention descriptions.

### The local adaptation of interventions is ignored

Health promotion practitioners argued that the ERS completely ignored that the interventions that they put to practice differed from the interventions that were developed and shown to be effective by others elsewhere. While most participants acknowledged the importance of adapting interventions to the local situation, there was disagreement about its consequences for the central rating of interventions. Some participants argued that adapting interventions invalidated effectiveness claims.

“*What exactly are you then assessing? You know by definition that something else will be implemented and it may be completely useless. That almost undermines the whole point of rating.*” (Policy-maker/committee member)

Other participants argued that, even though adapting an intervention limits the validity of an effectiveness claim, the likelihood that effects are realised remains higher because the working mechanism is still mostly the same.

“*So partly you end up with the same and partly you end up with something very different. It could well be that while the execution is very different, it still reflects the same underlying process which has been demonstrated to work at least that one time. You should not automatically assume that it will work just as well in a new setting; a lot could have gone wrong with that translation and so on. But it is at least promising.*” (Researcher/committee member)

Health promotion practitioners and researchers stressed that more attention must be paid to the adaptation of interventions and the implications this has for the validity of effectiveness claims. Health promotion practitioners were interested in examples of successful adaption and lessons learned during this process. They argued that the necessity of adapting interventions to the local situation implies that central rating and effectiveness claims can at best play a modest role.

## Discussion

The aim of this study was to explore the functioning of the ERS and the perspectives of researchers, policy-makers and practitioners regarding its contribution to improvement in practice.

The results show that the evidence base-inspired ERS approach was intended to function as part of a larger learning and improvement strategy in which researchers, policy-makers, funders and health promotion practitioners were expected to play a role. As part of this larger strategy, the ERS approach was intended to facilitate both the development of available interventions and the improvement of health promotion in practice.

The results show that, while participants expected that the describing and rating of interventions promoted learning and enhanced the transferability of interventions, they were concerned that the ERS approach was not suitable for steering and stimulating intervention development and improving health promotion in practice. The expert committees that assessed the interventions struggled with questions about how the effects of health promotion should be studied and what effects should be rated as relevant. Health promotion practitioners were concerned that the ERS neglected the local adaptation of interventions and did not encourage the improvement of aspects like applicability and costs, which they deemed important. Policy-makers and practitioners were worried that the lack of proven effectiveness legitimised cutbacks rather than learning and advancing health promotion. These results show that, while the measurement and central rating of the effectiveness of interventions can be beneficial, a narrow effectiveness-focussed evidence-based approach does not necessarily contribute to better health promotion in practice and may even hamper some of the processes that are required for its improvement.

An important strength of the ERS was that the process of carefully describing interventions, which was required for the first rating level, encouraged reflection among intervention submitters, better articulation of intervention components, and clarification of roles and responsibilities [8]. In addition, there were indications that the thorough descriptions enhanced the transferability of interventions and helped users opt for an intervention. The finding that carefully describing and naming interventions is useful is in line with analyses by Donald Schön, who has shown that such processes are key components of knowledge development by practitioners [19].

The limitations of the ERS emerged with the rating levels that focus on proven effectiveness. Those charged with determining if interventions were proven effective struggled with a lack of norms for what effects should be considered relevant, uncertainty about how health promotion works in complex open social systems and questions about how effects should be studied. These uncertainties, questions and challenges require attention, and show that the functioning of rating committees and the effective use of quality standards and empirical research is predicated on at least a partial agreement about normative and epistemic questions [12]. Put differently, an evidence-based approach can only work if the people involved agree about what effects are considered as relevant and how, by whom and when those effects can be established [20].

A problematic finding is that the ERS discriminates against the interventions that are considered most promising in health promotion. The research designs that top the hierarchy of evidence are primarily applicable to relatively simple, sequential interventions with a clear beginning and end, such as a medication that works through a tightly coupled biological or physical mechanism [21]. In health promotion, the greatest benefits are currently expected from interventions that simultaneously target the individual and their environment and comprise multiple components that are intended to be co-constructed in the local context [11, 22, 23].

Another finding that requires attention is that the ERS does not stimulate the improvement of aspects such as the applicability and costs of interventions. While a general description of these aspects was required for the first rating level, they were neglected in the subsequent rating levels. The hierarchy of evidence only stimulated the improvement of the proven effectiveness of interventions, whilst health promotion practitioners and policy-makers also need interventions that are cheaper, easier to realise, less demanding of context and more in line with the needs of target populations.

These findings suggest that the ERS (e.g. choice of committee members, forms for describing interventions, rating criteria) has a too narrow focus on effectiveness and neglects that interventions must be co-produced in diverse local situations. To better steer and promote the improvement of available interventions and health promotion in practice, a broader and more responsive approach is required, which takes into account the role of more actors and factors and knowledge from other sources than effectiveness research.

### The lure of effectiveness

The results show that the emphasis on proven effectiveness is a lure that is difficult to manage and can ultimately hamper the contribution to better health promotion in practice. This lure of effectiveness has at least four components. The first component is that, while the limitations of effectiveness claims are recognised, an image emerges that what is proven effective must be good, and vice versa. An intervention that is proven effective can still be impracticable, have additional negative effects or be too expensive. At the same time, the lack of proof of effectiveness does not mean that an intervention does not work. Effects may not have been studied or may emerge in a nonlinear way as a part of a confluence that does not satisfy the requirements of a standard experimental study design. A second component is the risk that the assumptions that interventions make about local contexts are neglected. An available intervention contains a ‘script’ full of assumptions about the local situation (e.g. resources, competences, support). These assumptions are often not well articulated, but essential for understanding what the effective intervention was comprised of and for realising a similar intervention elsewhere [12]. A third component is the risk that the work that is (and was) required to make an intervention successful is neglected. An available intervention is at best a hopeful design, and realising it locally in practice requires work by many actors and heterogeneous learning processes. Awareness that most of the work and learning required for realising an effective intervention has to happen locally is essential [24]. The fourth component of the lure of effectiveness is that an illusion can emerge that quality can be centrally assured. The quality of health promotion always depends on the local situation and can therefore never be centrally guaranteed [3]. Central quality assurance would require the same exact intervention to be realised at every location, which would seem impossible for the health promotion interventions that generally use a form of suggestion to try to influence anticipatory behaviour, which is deeply intertwined in a complex social context [12]. Instead of quality assurance, the more modest term of guidance seems more appropriate.

Similar questions about how to best employ research to contribute to improvement have been faced by those who develop guidelines for clinical medicine [7, 25]. In the early years of the evidence-based movement, recommendations were directly linked to the evidence for effectiveness. Soon, it became clear that various factors other than effectiveness (e.g. relevance, costs, side-effects) and the knowledge and experience of actors other than scientists (e.g. health workers, patients) had to be considered when developing recommendations [26]. Further reflection showed that high quality evidence should not necessarily lead to a strong recommendation, and a strong recommendation might be required even when the quality of evidence was low [27]. More recent approaches, such as the widely applied GRADE approach, therefore follow a two-step process, in which the quality of the evidence is first determined and a recommendation is then developed in a second stage [28]. By considering more factors and including more actors in developing recommendations, the GRADE approach seems better equipped to provide guidance to local practices. While GRADE has important strengths and can be used for inspiration, it however provides little guidance towards finding a socially robust way to determine which effects are relevant [29]. Furthermore, approaches like GRADE are not suitable for steering and promoting the process that should lead to the improvement of available interventions.

### Towards Responsive Guidance

**Fig. 2:**
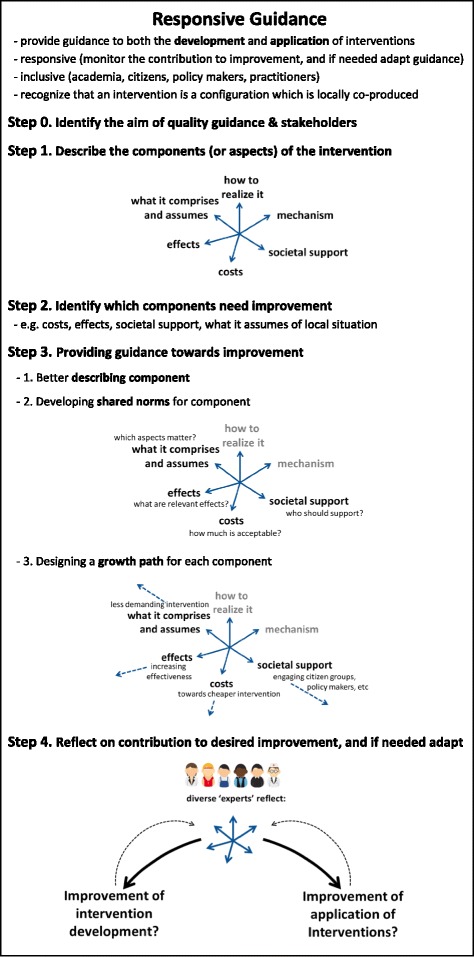
The steps of providing Responsive Guidance for achieving robust interventions

The results provide a number of insights that can be used to design an approach that is better capable of steering and promoting the improvement of available interventions and health promotion in practice. Such an approach, which we will refer to as Responsive Guidance, must assume that interventions are locally co-produced and that local learning is required for realising an intervention that works in local practice (Fig. 2).

What a ‘good’ intervention is depends on ever evolving local situations, ideas, norms and perspectives of the people involved. Instead of aiming for a universally applicable list of quality criteria, a more responsive approach is required that considers the situations in which interventions have to work. By analysing the needs and preferences of the involved actors and the functioning of local practices, it can be determined which aspects of an intervention (e.g. applicability, effectiveness, required resources) need improvement and what the direction for steering and promoting improvement should be. After deciding that a certain aspect of an intervention requires improvement (e.g. costs, effects), a first way to stimulate improvement could be to require that these aspects are carefully described (e.g. how much does it cost, which effects have been studied?); a second step could be to develop shared norms for an aspect (e.g. what effects are minimally relevant, what may an intervention cost?). Reflexive spaces need to be created in which norms (e.g. for relevant effects, acceptable costs) can be articulated and experiences with and results of interventions can be discussed and fed back to the development process [30–33]. To further steer and promote improvement, a growth path can be designed that provides further direction to the improvement of an aspect of interest.

The results indicate that, after rating interventions, the provider of central guidance should not sit back and hope that their ratings will be interpreted and used in a way that contributes to better health promotion in practice [34]. A range of efforts may be required to assist potential users with interpreting the provided guidance and discouraging the lure of effectiveness [35]. The need for more targeted guidance is recognised by the two-step approach of GRADE, in which simple and explicit recommendations are developed in the second step. Providing guidance for improving effectiveness does not necessarily require that interventions be rated as proven effective or not. A more modest alternative could be described, including, for each intervention, whether the effects have been studied and what effects have been found (in whom and in what circumstances).

What is further essential is to generate feedback loops from the practitioner and others who use the recommendations to the rating committees and from the rating committees to the intervention developers. By monitoring the way that recommendations are interpreted in practice, and gathering lessons about the use of interventions, the rating committees can adapt their guidance and can try to better steer and promote the development of new and existing interventions.

A challenge in public health is to develop knowledge that can be useful in multiple situations and thereby minimise the unnecessary duplication of research efforts [36]. The results indicate that the current focus on separate ‘interventions’ as a central unit of study requires enormous investments in research and may lead to overlap, as interventions with different names may consist of similar activities [37]. A more efficient way of employing research and learning may be to focus on mechanisms (or combinations thereof) instead of separate interventions [38]. Knowledge from research and practice can be gathered about these mechanisms and the way in which they produce certain effects in specific circumstances. Such an approach will require the development of a shared language for such mechanisms (and contexts and effects).

While this study provides relevant insights, it also has certain limitations. Ideally, we would have liked to follow the development, submission, assessment, use and improvement of several interventions over time. Conducting such a study would require intensive ethnography at multiple locations and be costly and difficult to organise. The strengths of this study are the large number and diversity of the individuals interviewed and the combination of interviews and observation of the actual functioning of the ERS committees.

## Conclusion

The aim of this study was to explore the actual functioning of the ERS and its contribution to improvement. According to participants, the ERS was expected to contribute to two separate, but linked improvement dynamics, namely (1) the improvement of available interventions and (2) the improvement of health promotion in practice. While participants expected that the describing and rating of interventions promoted learning and enhanced the transferability of interventions, they were concerned that the ERS approach was not suitable for guiding intervention development and improving health promotion in practice. The expert committees that assessed the interventions struggled with a lack of norms for the relevance of effects and questions about how effects should be studied. Policy-makers and health promotion practitioners were concerned that the ERS neglected the local adaptation of interventions and did not encourage the improvement of aspects like applicability and costs. They were also worried that the lack of proven effectiveness legitimised cutbacks rather than advance health promotion. To better contribute to improving health promotion, a more responsive guidance approach seems to be required, namely one which stimulates the improvement of different intervention aspects, is more specific about the meaning of the recommendations provided and provides feedback to those who develop and rate interventions. To contribute to improvement, such responsive guidance must always be embedded within a broader learning and improvement strategy, which must include regular reflection on whether that overall strategy contributes to better health promotion and ultimately better health.

## Abbreviations

ERS: effectiveness rating system
