# Correction to: Improving health promotion through central rating of interventions: the need for Responsive Guidance

**DOI:** 10.1186/s12961-018-0294-0

**Published:** 2018-03-08

**Authors:** Maarten Olivier Kok, Roland Bal, Caspar David Roelofs, Albertine Jantine Schuit

**Affiliations:** 1 Erasmus School for Health Policy and Management, Erasmus University Rotterdam, Burgemeester Oudlaan 50, 3062 PA Rotterdam, The Netherlands; 2 Amsterdam Public Health Institute, VU University, Amsterdam, The Netherlands; 3 Science and Society, Faculty of Mathematics and Natural Sciences, University of Groningen, Groningen, The Netherlands; 4 National Institute for Public Health and the Environment, Bilthoven, The Netherlands

## Correction

It has been highlighted that the original manuscript [1] contains a typesetting error in the name of Caspar David Roelofs. This was incorrectly captured as Caspar David Roelefs in the original manuscript which has since been updated.

## Affiliations

1. Department of Gynecology and Obstetrics, Goethe-University, Frankfurt, Germany.

2. Division of Preventive Medicine, Institute of Occupational Medicine, Social Medicine and Environmental Medicine, Goethe-University, Frankfurt, Germany.

3. Department of Obstetrics and Gynecology, Keck School of Medicine, University of Southern California, Los Angeles, CA, USA.

4. Department of Preventive Medicine, University of Southern California, Los Angeles, USA.
